# *Brighton v RSPCA NSW*: Appeals and Lessons Four Years On

**DOI:** 10.3390/ani14223345

**Published:** 2024-11-20

**Authors:** Kathryn Jurd, Sophie Riley

**Affiliations:** 1 RSPCA NSW, Yagoona, NSW 2199, Australia; 2 Faculty of Law, University of Technology Sydney, Ultimo, 2007 NSW, Australia

**Keywords:** animal law, serious animal cruelty, criminal intention, statutory interpretation, animal cruelty prosecution, animal cruelty litigation, full subjective intention

## Abstract

Animal law has the potential to initiate improvements for animal wellbeing. However, this largely depends on how effectively the law bridges the legal chasm between animal welfare and animal suffering, a chasm the authors refer to as the welfare gap. When the law does not adequately address this gap, where regulation subordinates animal interests to human interests, it results in weak animal protection that does little more than regulate to a standard that avoids a life not worth living. The authors analyse a series of cases involving the RSPCA and Brighton, in which Brighton was charged with serious animal cruelty pursuant to s 530 of the Crimes Act 1900 (NSW). He stabbed a dog with a pitchfork; after failing to kill the dog, he suspended it from a tree branch by a leash attached to its collar and struck the dog several times on the head with a mallet, finally killing him. Brighton was found guilty in the NSW Local Court and appealed to the NSW Supreme Court, where Rothman J allowed the appeal, holding that Brighton had exterminated a pest animal. This led to protracted litigation, including to the NSW Court of Appeal, a second hearing in the Local Court and a further appeal to the Supreme Court. In August 2020, Sophie Riley published a case note and commentary on the litigation up to the Rothman J appeal. This paper evaluates the litigation that followed, identifying how regulatory failures have entrenched the welfare gap. Regulatory failures include inadequate and aged legislative protections for a confined subset of animals. In NSW, animal sentience is not enshrined in legislation; the law limits the types of animals protected by anti-cruelty law; fundamental statutory language remains undefined, for example terms such as “pest animal” and “exterminate”; and challenges abound for adducing sufficient evidence to prove subjective criminal intent. These deficiencies pose significant challenges for practitioners and judicial officers, particularly when complex statutory interpretation is required in the busy and fast-paced summary jurisdiction. This paper concludes that legislators should consider modernising the law, removing ambiguity, and settling minimum standards for a good life for animals, taking into account the welfare aspects described in Mellor’s Five Domains model.

## Simple Summary

On 14 January 2016, Daniel Brighton killed one of two dogs that had strayed onto his property, where they had attacked Alice, a camel owned by a Petting Zoo business operated by Brighton. In late August 2017, the Royal Society for the Prevention of Cruelty to Animals, New South Wales (RSPCA) became aware of the allegations, commenced an investigation, and thereafter preferred charges in the NSW Local Court at Campbelltown. As was his right, Brighton pleaded not guilty to two charges, brought pursuant to s 530 of the *Crimes Act 1900* (NSW), which alleged that he committed acts of serious animal cruelty. The charges particularised offences that, whilst intending to inflict severe pain on the dog, Brighton firstly seriously injured the dog by stabbing him with a pitchfork several times and secondly, that he subsequently killed the dog by hanging him from a tree and striking him with a mallet. Brighton maintained his not guilty position until 13 December 2023, when during the hearing of the second of two Supreme Court Appeals, before Hamill J, he withdrew that appeal (against the conviction), as well as a concurrent appeal in the District Court (also against conviction and sentence). The Hamill J decision was published on 12 January 2024, two days shy of the eighth anniversary of the attack on Alice and the dog’s slow and painful death.

## 1. Introduction

On 12 January 2024, Justice Peter Hamill SC published the final judgment in respect of proceedings which had commenced more than six and a half years earlier [1]. That judgment related to charges brought by the Royal Society for the Prevention of Cruelty to Animals New South Wales (RSPCA) contrary to s 530 *Crimes Act* 1900 (NSW) [2]. The first nearly three years of these proceedings were considered in Sophie Riley’s original consideration of this matter [3] and this article follows the litigation following the first Supreme Court decision by Justice Rothman relating to the two serious animal cruelty charges [4]. 

Those charges related to an incident on 14 January 2016, where Brighton killed one of two dogs that had strayed onto his property, after they had attacked Alice, a camel owned by a Petting Zoo business which Brighton operated. Brighton caught and then killed one of the dogs, initially inflicting non-fatal injuries by stabbing the dog with a pitchfork. Brighton then suspended the dog in a tree via a leash attached to the dog’s collar and struck the dog five or six times with a mallet. The cause of death remains unknown, although likely asphyxia, as none of the observable injuries to the skeletal remains were sufficient to cause death alone or in combination with each other [5]. The RSPCA became aware of the allegations in August 2017 and after investigating the matter, preferred charges against Brighton, in the NSW Local Court at Campbelltown [6]. On 17 June 2019, Brighton was found guilty by Magistrate McAnulty, setting in motion a series of appeals and counter-appeals that only ended in December 2023, when Brighton withdrew his conviction appeal and, for the first time, accepted his guilt for the offences charged.

In August 2020, Riley published a case note and commentary on the first of these appeals, which was made by Brighton against his initial conviction [7]. The case was heard by Rothman J SC in the Supreme Court of New South Wales on 3 April 2020 where his Honour allowed the appeal [8]. In the case note and commentary, Riley argued that a contextual rather than textual approach to statutory interpretation would have been more appropriate in the case. Riley additionally argued that the legal chasm between anti-cruelty regulation and the legal formulation of animal welfare concepts means that animals are not well protected within the law [9].

The purpose of this article is to provide updated commentary and analysis on the subsequent litigation, up to the final appeal, which was to be heard in the NSW Supreme Court by Hamill J SC on 13 December 2023 [10]. Brighton had steadfastly maintained his innocence up until the time he withdrew his conviction appeal before Hamill J SC. Consequently, whilst convictions were recorded against him, this occurred without a final appellate determination of the case against him. Possibly more importantly, however, the withdrawal of the appeal avoided the necessity of additional interpretation from the NSW Supreme Court as to the operation of the serious animal cruelty offence in the Crimes Act. As a result, and given the complex judicial course this matter took, the arguments made by Riley in 2020 with respect to the difficulties faced by judges in translating animal welfare protections into a legal concept remain.

Amongst other things, this article argues that by failing to enshrine sentience, and without specific statutory requirements which require minimum standards for the treatment of all animals under the influence of humans, the law in NSW creates a legal chasm that does little more than regulate to a standard that hopes to avoid a life not worth living. The authors refer to this chasm as the welfare gap. The article commences with a detailed chronology of the proceedings, which provides important background for analysing the decisions and associated implications for animal welfare. The authors concentrate on three topics: the importance of statutory interpretation in anti-cruelty regulation; the difficulties of proving serious animal cruelty based on the subjective intention of a defendant; and how law and policy create and entrench the welfare gap. It is concluded that to bridge this gap, statutory amendment is required that addresses these issues and should integrate domain theory as proposed by Mellor [11].

Before commencing the discussion, the authors make two comments on changes to the law that have occurred throughout the course of the various proceedings, but which did not apply to the defendant. First, from 17 June 2021, the statute of limitations for offences brought under the *Prevention of Cruelty to Animals Act 1979* (NSW) (POCTAA) increased from 12 months from the commission of the offence to three years after the date evidence of the alleged offence first came to the attention of an officer [12]. This means that had the events occurred today, charges could have been preferred under POCTAA, where offences are strict liability, and as such, do not require proof beyond reasonable doubt of the subject intention of the defendant. Second, on 3 July 2017, section 530(1A) was introduced into the *Crimes Act* [13], to permit charges to be preferred where there is proof beyond reasonable doubt that the defendant acted recklessly as to the infliction of severe pain to the animal, while beating, torturing, or killing the animal.

## 2. Chronology of Proceedings

As the introduction to this article foreshadows, the litigation in this matter spanned some six years, commencing in 2017 with the laying of charges in the Local Court at Campbelltown and ending with the final appearance of the parties in the NSW Supreme Court in December 2023. The case generated some 65 court appearances. Readers of the original article [7] will recall that it dealt with litigation to the point of the successful appeal by Brighton in the Supreme Court before Rothman J [14]. The current article considers the course of this matter in the four years since that appeal. Briefly, the RSPCA appealed the Rothman J decision to the NSW Court of Appeal [15], where the successful appeal meant the case was remitted to the NSW Local Court, followed by a second sojourn in the Supreme Court of NSW. Table 1 sets out a precis of the litigation, which is elaborated below.

### 2.1. First Local Court Hearing

On 13 October 2017, the RSPCA commenced proceedings against Brighton in the NSW Local Court at Campbelltown alleging that he committed two offences of serious animal cruelty contrary to s 530(1) *Crimes Act 1900* (NSW) [16]. That section provides:

(1) A person who, with the intention of inflicting severe pain—
  (a) Tortures, beats or commits any other serious act of cruelty on an animal and
  (b) Kills or seriously injures or causes prolonged suffering to the animal is guilty of an offence.

Section 530(1A) (added in 2017) creates a sub-category of the serious animal cruelty offence whereby recklessness as to the infliction of severe pain is substituted as the mens rea (mental element) of the offence.

The elements of the s530(1) offence have been summarised in Table 2 as follows:

There has been some disagreement as to whether it can properly be said that section 530(2) operates as a defence, as distinct from an element of the offence, the negative of which must be proved beyond reasonable doubt. Justice Rothman declined to categorise the provision further [19]. The preface of the provision provides “[a] person is not criminally responsible” if, amongst other things, the conduct occurred “in the course of or for the purpose of… the extermination of pest animals…” indicating, to some minds, that the provision should be interpreted as an element rather than a defence.

However, in the first appeal, Rothman J referred to the section as a “so-called defence”, casting doubt on its true nature [20]. On this question, in the Court of Appeal, President Bell (as his Honour then was) commented “the better view, in my opinion, is that s 530(2) operates as a defence and the onus of establishing it lies with the defendant.” [21] This is the view that the parties had adopted at first instance, and then again in the Supreme Court, more specifically that once the defence was raised on the balance of probabilities it then needed to be negatived beyond reasonable doubt by the Prosecution. As the Court of Appeal ultimately determined that, as a matter of statutory construction, section 530(2) operates as a statutory defence, the section is so described in this article [21].

Throughout the hearings, the RSPCA argued that, whilst intending to inflict severe pain on the dog, Brighton seriously injured the dog by stabbing him several times with a pitchfork (sequence one before the court), and seriously injured or caused prolonged suffering of the dog. In the second sequence, the RSPCA argued that, with the requisite intention, Brighton seriously injured the dog by suspending the dog from a tree, striking him with a mallet and ultimately killing the dog [22]. More than seventeen months later, the hearing proceeded over two days on 28 and 29 March 2019. The evidence included expert evidence from a veterinary forensic pathologist from Taronga Zoo who examined the remains of the dog [23]. The facts, which, from the NSW Supreme Court proceedings onwards, were not disputed by the defendant, are summarised by Rothman J at paragraphs 11–19 of his 2020 decision and accept the findings with respect to the conduct of the offending, as found in the Local Court [23]. This material makes for distressing reading, but prior knowledge of the facts is assumed throughout much of this article.

At first instance the matter was heard before Magistrate McAnulty, where Brighton raised the defence found in section 530(2), that he was exterminating a pest [24]. Magistrate McAnulty declined to find the statutory defence made out and entered guilty verdicts with respect to both offences sentencing the defendant to an aggregate term of imprisonment of 3 years and 4 months, which included a non-parole period of 2 years and 2 months [25].

### 2.2. First Appeal to the Supreme Court

On 26 June 2019, Brighton appealed the conviction and on 9 September 2019, he also appealed the severity of the sentence. The appeal for both matters was heard on 3 April 2020 by Justice Rothman J, who delivered the decision on 23 April 2020 [14]. As readers of the original article will recall, this matter was decided on the basis of an error in the Local Court, where the Magistrate had used a contextual analysis of the legislation and which furthermore, precluded the defendant from relying on the statutory defence of “extermination of pest animals” [26]. The Crimes Act does not contain a definition of either a “pest animal”, or “extermination”. Rothman J acknowledged that words and their definitions “turn on the context” but nevertheless relied on dictionary definitions in order to interpret the statutory defence by deconstructing it into its constituent parts: “extermination” and “pest animal(s)” [27]. The Magistrate had used a more openly contextual approach, however Rothman J held that the Magistrate did not interpret the legislation according to law, consequentially he upheld the appeal [28].

### 2.3. Second Appeal, to the Court of Appeal

On 22 May 2020, the RSPCA appealed to the NSW Court of Appeal on the grounds that Rothman J erred, first, in finding that the dog was a “pest animal” and second, that Brighton’s actions amounted to an “extermination” of a pest, in accordance with s 530(2) of the Crimes Act.

The appeal was heard on 8 December 2020, before Bell P, Basten JA and Simpson AJA, with a reserved judgment published on 23 December 2020 [29]. Each Judge agreed with orders proposed by the President, which, subject to one issue, relating to proof available with respect to the subjective intention of the defendant (see Section 3.2, below), remitted the matter for redetermination to the NSW Local Court [30].

Thus, the issue of statutory interpretation loomed large.

Justice Basten observed that “[t]he applicant (the prosecutor in the Local Court) submitted that the scope of the defence should be determined by treating the phrase “extermination of pest animals” as a composite phrase to be understood in its specific statutory context.” [31]. His Honour agreed with this approach, noting that the use of dictionary definitions “was conducive to error” because it took the Court’s attention away from the statutory context [31]. Arguably, this also results in what might be described as “duelling definitions” (the authors’ phrase, not his Honour’s), when such a (dictionary) definition could not assist in the present case.

President Bell held that in killing the dog, Brighton was not exterminating a pest animal. Although his Honour agreed that in the circumstances, the dog was a pest animal, he indicated that exterminating a pest normally refers to a systematic approach that aims to eradicate as many pests as possible from the one process of extermination [32]. This is not what the defendant did. His Honour extended the evaluation by referring to section 22 of the *Companion Animals Act 1998* (NSW) [33], which permits the destruction of an uncontrolled dog in limited circumstances, and subject to several requirements, including those found in sections 22(9) and 22(10). However, as is made clear in section 22(9), whilst an occupier of land may take action to destroy a dog if they reasonably believe the dog will molest, attack, or cause injury to animals, they are not permitted to contravene POCTAA in so doing. Additionally, the dog must be killed quickly “and without unnecessary suffering” [34]. Again, given the manner of killing, this statutory context reinforces the Judge’s conclusion that the statutory defence had not been made out.

Acting Justice Simpson largely agreed with the findings of President Bell but disagreed that the dog was a pest animal [35]. Her Honour also agreed that extermination “carries a connotation of mass removal” [36]. Accordingly, to be a pest animal, “an animal must belong to a class of animals that can be categorised as ‘pests’” [37]. Her Honour’s reasoning concludes with the persuasive determination that “a single act of attacking the camel was insufficient to establish” the dog as a pest animal [38]. The Court of Appeal ultimately found that the use of dictionaries in Brighton v Will did not aid either the Local Court, nor the Supreme Court in construing the legislation and moreover, as a matter of statutory interpretation the defence was not made out [39].

Accordingly, the RSPCA appeal was allowed. Notwithstanding this, the Court also held that that the Magistrate at first instance failed to make explicit findings as to the specific intention required, as an element of the s 530(1) offence. It will be recalled that the section commences with the phrase “[a] person who, with the intention of inflicting severe pain…”. The issue turns on what is meant by the word “intention”, and the impact of different interpretations of that word on the outcome of the case. The three Judges ultimately agreed that the decision did not make explicit the evidentiary basis for the determination that full subjective intention had been proved beyond reasonable doubt. Consequently, the matter was remitted to the Local Court of NSW for re-determination according to law, and the orders of Rothman J were set aside [40].

### 2.4. Second Local Court Hearing

The second Local Court hearing occurred at Campbelltown Local Court, before Magistrate Degnan, between 15 and 17 December 2021 [41]. The evidence before the Court included the transcript from the first proceedings, as well as the exhibits tendered before Magistrate McAnulty. In addition, the forensic pathologist who conducted the necropsy was recalled to give evidence on the efficacy of the methods deployed by the defendant to kill the dog.

On this point, the sole issue for determination was whether the RSPCA had discharged its burden to prove beyond reasonable doubt that Brighton possessed the full subjective intention required by s530(1). The authors will revisit this point in Section 3.2 below. However, at this stage, it is worth noting that the RSPCA argued that there was sufficient evidence that the defendant understood the inevitable consequence of conducting himself as he did [42]. Moreover, that understanding, taken in conjunction with other inferences rationally drawn, for example stabbing a dog with a pitchfork, leaving the pitchfork embedded in the dog for up to 45 minutes, then hanging the dog from a tree whilst beating it with a mallet, was behaviour consistent with an intention to cause severe pain to the dog, motivated, as he was by anger and retribution toward the dog for the admittedly vicious attack on Alice the camel. The defence, conversely, argued that there were inferences to be drawn, consistent with innocence, which meant that the Prosecution had not properly discharged its onus.

On 8 February 2022, Magistrate Degnan delivered a judgment in which he found that the offences had both been proved beyond reasonable doubt, and adjourned the sentence proceedings to 11 July 2022 [43]. With respect to the issue of intent, his Honour found that the Prosecution had proved the element beyond reasonable doubt with respect to each charge [44]. On 7 March 2022, and prior to the sentencing hearing, the defendant appealed the conviction to the Supreme Court of NSW. In the meantime, the sentence proceedings took place in the Local Court as listed and Brighton was convicted of the two section 530(1) offences and sentenced to imprisonment for two years and two months, with a non-parole period of two years [45].

### 2.5. Second Supreme Court Appeal

As indicated above, the second Supreme Court Appeal was initiated by Brighton on 7 March 2022. More than four months later, on 26 July 2022 Brighton added a further ground of appeal, arguing that the sentence was manifestly excessive [46]. The matter was listed for hearing in the Supreme Court of NSW on 31 May 2023, before Hamill J. On that day, Brighton sought an adjournment and leave to file an amended summons, both of which were granted, and the matter was relisted for hearing on 13 December 2023.

The matter proceeded to a hearing, when Brighton ultimately withdrew his appeal against the conviction, leaving in place his appeal against the severity of the sentence. Hamill J made orders dismissing the conviction appeal, granting leave to appeal against the sentence, and imposed an aggregate two-year term of imprisonment to be served by way of intensive correction order pursuant to section 7 of the *Crimes (Sentencing Procedure) Act 1999* (NSW) [47]. On 12 January 2024, two days shy of eight years after Brighton killed the dog. Hamill J published the judgment in this matter [48].

## 3. Analysis

Readers will no doubt agree that the litigation in this case was protracted and convoluted. In addition, the conclusion of the matter, in which Hamill J imposed an intensive corrections order, a sentence which had first been indicated by Rothman J some four years earlier, is somewhat unsatisfactory because the opportunity for Hamill J to provide additional judicial commentary on the operation of section 530 was denied. Consequently, at least three important issues remain for consideration: statutory interpretation with respect to the intention elements and defences set out in section 530; the challenges of proving full subjective intent in animal cruelty cases; and the role of animal welfare in anti-cruelty regulation. The first and third issues were dealt with in Riley’s original article and are, here, the subject of further evaluation and analysis. The second issue, that of intent, was identified by the Court of Appeal and is examined in this article for the first time. The material is then drawn together in a discussion of animal welfare, concluding that law and policy, at least in NSW, have not adequately bridged the gap between the theory and its practical application, calling for a transformative approach, such as domain theory proposed by Mellor.

### 3.1. Statutory Interpretation

In her article, Riley suggests that a contextual approach, rather than a textual one might have served the statutory analysis better. Statements by Basten JA and Bell P, in the Court of Appeal, endorse that approach, and indeed the contextual analysis applied by the Court of Appeal led to the defendant not being able to rely on the defence provided by section 530(2) [49]. As discussed above, Basten JA noted that applying dictionary definitions to each word or phrase, such as “pest animal” and “extermination”, was conducive to error, with Bell P pointedly indicating that using dictionary definitions should not replace “the ordinary process of statutory construction” [50]. Both judges agreed that statutory interpretation should occur “in the broader context of the relevant provisions…” [51]. This finding is consistent in earlier cases, where the High Court had similarly held that “[t]he starting point for the ascertainment of the meaning of a statutory provision is the text of the statute whilst, at the same time, regard is had to its context and purpose” [52]. The emphasis on context is important for a number of reasons.

Primarily, dictionaries invariably proffer a range of meanings, but without direction as to the most appropriate choice of meaning within the framework of the legislation [53]. Section 530 does not define the terms “pest animal” or “extermination”, so the Court of Appeal turned to the second reading speech for guidance [54]. The section was introduced into the NSW Parliament in 2005 as the Crimes Amendment (Animal Cruelty) Bill [55] with the second reading occurring on 9 and 15 November 2005 [56].

Both the second reading speech and subsequent debate emphasised that the Bill was the outcome of recommendations made by a multi-agency Animal Cruelty Task Force that had been established by the Government in response to community disgust at animal cruelty [57]. Examples that informed the work of the Task Force included a defendant beating dogs and puppies to death with a brick [58] and another incident where a person threw a kitten onto a railway track [59]. The Task Force recommended that the Government introduce a new offence for those who torture, beat, or commit any other act of serious cruelty against an animal with the intention of inflicting severe pain [60]. The issue of intention was to be central to the new offence, with the accused afforded an opportunity to show that they lacked “the requisite intent” and instead hurt the animal accidentally [61]. In addition, it is clear from the second reading speeches that the new legislation was to operate additional to, but within the framework of existing regulation, including POCTAA and the *Animal Research Act 1985* (NSW) [60].

Clover Moore and David Barr, in the Legislative Assembly, also discussed society’s relationship with animals, noting that humans are close to their companion animals, but also acknowledging that some animals are treated cruelly [62]. More specifically, David Barr observed:

In all aspects of our relationship with animal species we should accord animals the dignity they deserve and respect their right to occupy a place on this planet. We do not have a monopoly on the right to dignity and a reasonable lifestyle; animals should be entitled to live out their lives in an urban environment and in their natural habitat [62].

President Bell similarly noted that amendments to the Crimes Act had been triggered by community abhorrence at unprovoked attacks on animals [63], and that the “new” (now nearly 20 years old) legislation was designed to deal with heinous acts of animal cruelty as a way of supplementing existing law and policy, not replacing it [63]. These findings reinforce that a contextual approach to interpreting section 530 is preferable to a textual one, a stance further underscored by the Court of Appeal’s interpretation of “pest animal” and “extermination”.

As already indicated, the phrase “pest animal” is not defined in section 530. Turning to a dictionary may not be helpful because, as the Court of Appeal observed,

the same species of animal may be regarded in different ways, drawing examples such as kangaroos that are managed as a pest species in agricultural areas, notwithstanding their status as a national icon. Similar debates could arise with respect to eagles, reptiles, native species generally, and foxes [64].

Yet, the attractive simplicity of this logical conclusion might be muddied by the type of animal in question and the regulatory context. In Queensland for example, a fox is a restricted invasive animal pursuant to the *Biosecurity Act 2014* (QLD) [65]. In accordance with that Act, it is not possible to keep a fox domestically, nor is a person able to feed a fox, or move it [66]. In these circumstances, if a defendant were to argue that they were exterminating a pest, it is difficult to see how the behaviour of the animal would be relevant. Conversely, is it possible to argue that a dog, which can be a companion animal, can be a pest because of its behaviour? [67].

Arguably, the consequence of this statutory imprecision, in combination with strict textual analysis, may lead to an increase in the number of animals, and contexts within which animals could be, classified as a pest. It also leaves open to individual judicial interpretation the nature of the animal that has been tortured, killed, injured, or caused to suffer without fear of criminal sanction. That is, animals more obviously companion, or less obviously “pest-like”, may attract greater protections in circumstances where arguably the point of such provision should be to protect all animals capable of suffering, from suffering, occasioned at the hands of humans [68]. To interpret the section otherwise clearly appears contrary to the intention of the legislation. In Section 3.3 below, the authors discuss the reluctance of the NSW legislature to enshrine animal sentience in the criminal law. Even leaving aside the gaps in animal protection that this omission creates, it is difficult to countenance provisions which could be interpreted to permit egregious animal cruelty based on the type of animal to which the charge attends itself. One saving grace is the way the Court of Appeal interpreted the word “exterminate”.

Basten JA agreed that the phrase “extermination of pest animals” should be interpreted in its statutory context and should also be treated as a composite phrase [31]. He noted that the dictionary definition of this word meant to “destroy utterly” or “get rid of” and this was not helpful in the present case where one dog was targeted and killed [69]. The killing of individual pest animals is designed to reduce population numbers, but not necessarily eradicate populations [70]. In a practical sense, extermination refers to “a systematic and regulated approach to dealing with an identifiable problem” [69]. Accordingly, a person who undertakes an official control program would be protected by the defence, but someone acting individually and unauthorised, would not be so protected [71]. As Brighton was acting individually and not authorised, he was not exterminating a pest and hence, could not rely on the defence.

This analysis accords with Riley’s arguments in the original article, and indeed applying a contextual approach to statutory interpretation led to Brighton not being able to rely on the defence set out in section 530. In a practical sense, a contextual interpretation prevents the defence being used to support a spur of the moment and deliberate reaction that results in serious animal cruelty. The court itself held that to find otherwise would be incompatible with the “balance” sought to be struck by parliament and would also “defeat or at the very least undermine the legislative purpose underpinning section 530(1)” [33]. Where the act is accidental, though, different considerations apply, because if truly accidental it could not meet even a recklessness test.

### 3.2. Intention and Serious Animal Cruelty Cases

The elements of section 530 include a requisite mental element—namely that the defendant with “the intention of inflicting severe pain”, tortures, beats, kills, seriously injures or causes prolonged suffering to the animal. There is no doubt the section requires evidence of specific intent. The question, however, is to which element must there be this coincidence between mens rea (guilty mind) and actus reus (guilty act) and is it sufficient to prove an objective or a subjective state of mind? In the former case, the intention to commit the acts, for example, beating an animal, without intending to torture, seriously injure, or cause prolonged suffering would arguably be sufficient to trigger the legislation. On the other hand, a full subjective intention, requires a “deliberate subjective mindset” to torture and/or cause prolonged suffering [72]. Bell J concluded that the legislation refers to subjective intention [73] and this is in “keeping with the higher penalties” imposed under the Crimes Act compared to POCTAA [74].

Without doubt this approach is consistent with the second reading speech, discussed above, which draws a distinction between deliberate acts and accidentally injuring or killing an animal. It should also be kept in mind that sections 5 and 6 of POCTAA deal with cruelty and aggravated cruelty that captures conduct envisaged by section 530, and these offences are strict liability offences [75]. However, readers familiar with the NSW statutory scheme will be aware that POCTAA offences attract significantly lower penalties compared to the Crimes Act.

The requirement to prove, beyond reasonable doubt, the full subjective intention at the time of the conduct which comprises the serious animal cruelty, makes this offence more difficult to prove than its strict liability cousin in POCTAA. At the same time, and unlike the section 530 offence, the strict liability offences in POCTAA have available a common law defence of honest and reasonable mistake of fact [76]. The expiry of the limitation period pursuant to section 34(4) of POCTAA, makes this point relatively academic with respect to the subject case. Accordingly, there were no POCTAA offences capable of being preferred, nor did the section 530(1A) reckless alternative (discussed in the introduction) exist at the time.

The inevitable consequence of the foregoing is that the law has created a paradox, contrary to the tenor of the second reading speech accompanying the introduction of section 530. To start with, as is obvious given the criminal jurisdiction, this section requires proof of every element of each charge beyond reasonable doubt. Consequently, had the Court been persuaded, for example, that with respect to sequence two (killing the dog or causing prolonged suffering to the dog), the defendant intended to kill the dog to put it out of its misery, or even intended to hand out some punishment for the injury to Alice, but did not intend to inflict severe pain, that would have been sufficient to defeat this charge.

In the judgment published after the second Supreme Court Appeal, Hamill J accepted by virtue of the withdrawn appeal, that “[o]nce it is accepted that the plaintiff had the requisite intention, there is little doubt that the offences were extremely serious offences of their kind and exhibited a degree of barbarity at odds with the plaintiff’s previous good character and history of employment in caring for animals” [77]. His Honour noted that of relevance to the objective seriousness (which must be determined prior to sentencing), that the issue was not just the overt attacks on the dog, but the failure thereafter to ensure the dog was dead, and the periods of time during which it was left to endure pain. Furthermore, Hamill J accepted, beyond reasonable doubt, “that that the plaintiff acted in an angry rage and was seeking vengeance against the dog” [78].

There remains, however, an outstanding question as to whether the community and Parliament, as the community’s elected representatives, intended the death of animals to be inflicted legally in this manner. This question brings the discussion to the welfare gap and the role of law and policy in its creation and continuation.

### 3.3. The Welfare Gap

Recently, questions have been asked as to how well POCTAA performs in preventing animal cruelty in NSW [79,80]. It is evident that in many respects, this central piece of legislation, which should be focused on improving the lives of animals, is outdated, has not benefited from piecemeal amendment over the 45 years since its introduction, and does not adequately achieve good lives for animals [11]. In reality, POCTAA does little more than attempt to regulate to a standard that attempts to avoid a life not worth living and could be improved by legislating towards a minimum standard for achieving a good life for animals.

In the original article, Riley identified a number of ways in which the legal chasm between acceptable animal welfare and animal suffering has become entrenched [7]. Broadly speaking, issues revolve around how regimes “integrate law, science and ethics” and the sway this integration holds sway over the introduction of law and policy, as well as the interpretation of legislation.

One basic example derives from the wording of section 530 and the use of the word “torture”. In animal cruelty cases, the nature of the offending is critical. The word torture is arguably difficult to define and lacks the type of specificity that avoids unscientific emotion. It is doubtful, for example, whether expert evidence could be adduced on whether an animal has been tortured. For this reason, the Prosecution of the cases against Brighton relied on the element of prolonged suffering, as it was more feasible to obtain veterinary evidence on this matter. There is now an abundance of scientific and scholarly literature to prove that a wide range of animal species are sentient [81,82,83]. This means they have the capacity to experience positive and negative feelings such as pleasure, joy, pain and distress that matter to them as individual animals [84]. From an animal welfare perspective, it is thus important for legislation to be clear, both about the animals it is protecting, and the nature of the offending because this forms the basis of anti-cruelty regulation and the offence provisions themselves.

However, even an issue as basic as what an animal is can become contentious because, once an animal is brought within the parameters of statutory definitions, they receive the protection of the law. This situation brings the law face to face with societal expectations and preconceptions as to the types of animals that should be protected.

Section 4 of POCTAA, for example, defines an animal as (a) any vertebrate, including amphibians, birds, fish, (non-human) mammals, and reptiles, or (b) a crustacean, but only when being prepared as food. The latter is an attempt to prevent cruelty to crustaceans by boiling or butchering them alive and was added to the definition of an animal in POCTAA in 1997. Section 530(3) of the Crimes Act, however, defines an animal as a mammal, bird or reptile. This restricts the animals that are afforded protection against serious animal cruelty by excluding crustacea, fish and reptiles. In 2016, the RSPCA issued a penalty infringement notice, pursuant to POCTAA, to a fishmonger at the Sydney Fish Markets for committing an act of animal cruelty on a lobster, which was dissected alive, on a bandsaw. Macquarie University academic, Associate Professor Culum Brown gave an opinion, permissible in accordance with section 79 of the *Evidence Act 1995* (NSW) as to the proven capacity of crustacea to feel pain [85] and the defendant company was convicted and fined $1500 AUD following an appeal to the NSW District Court [86]. There is no doubt that the fishmonger determined to kill the lobster, there is furthermore, no doubt that dissecting the lobster with a bandsaw would cause the lobster to suffer, and yet without proof that the defendant subjectively intended to inflict severe pain and suffering, a Crimes Act offence is unlikely to have been successful.

In practice, statutes reflect the fact that legal regimes identify a level of cruelty that society is prepared to tolerate [87]. This is consistent with the utilitarian underpinnings of animal welfare, where the probity of an act or omission is determined by its consequences, taking into account the parties’ interests [88]. Accordingly, law and policy weigh animal sentience against human interests, invariably subordinating the former to the latter [89,90].

In the same vein, anti-cruelty regulation contains many exceptions to proscriptions against animal cruelty. As such, definitions of cruelty found in section 4(2) of POCTAA refer to cruelty in terms of acts or omissions that have “unreasonable, unnecessary or unjustifiable” consequences, while section 24 of POCTAA provides exceptions for stock animals and animals in research.

The contentious nature of defining an animal, recognising animal sentience, and the impact of this recognition on anti-cruelty regulation and animal welfare, is underscored by the circumstances surrounding two pieces of draft legislation that were released for comment in New South Wales in 2022. Before discussing the draft legislation, the authors note that the term animal welfare does not, on its own, provide any indication of the minimum welfare standard to be protected. If regulation is to promote animal welfare it needs to be promoting it to specified minimum standard of good, very good, or otherwise.. In addition, up until this point NSW has declined to refer explicitly to animal sentience either within the objects of existing legislation or in relation to proposed legislation [91]. It is telling that regulators have not explained what risk is ameliorated by excluding a legislative reference to sentience.

The first proposed legislation, the Animal Welfare Bill 2022 (NSW) was prepared by the NSW Department of Primary Industries and Regional Development (DPI) and released for public comment on New Years Day in January 2022 [92]. The objective of the bill ostensibly remains promoting welfare, but it does not mention animal sentience. The bill was never tabled in parliament, leading Abigail Boyd MLC, in August 2022, to introduce a private members bill, the Prevention of Cruelty to Animals Amendment (Animal Sentience) Bill 2022, into the upper house of the NSW Parliament [93]. Although the Animal Sentience Bill adds sentience as a concept, it does not appear to change the definition of animal [94]. During the second reading speech of this Bill, in August 2022, Senator Boyd had this to say about the DPI’s earlier Animal Welfare Bill:

At the beginning of this year, the then Minister for Agriculture referred this draft (Animal Welfare) bill to a parliamentary inquiry, presumably knowing that the committee could never reach a consensus on the need to update our laws because of the diversity of views—however outdated and misguided some of them may be—of the members that make up the committee [94].

In any event, the Animal Welfare Bill did not proceed and lapsed on 27 February 2023.

Returning now to the earlier Animal Welfare Bill 2022, three issues are telling: first, the Bill attempts to set minimum care requirements, however they are not directed at improving animals’ lives, and thus the Bill does not legislate for lives worth living for animals, in accordance with the domains model; second, the legislation was never tabled in Parliament, again demonstrating the controversial nature of animal welfare reform; third, the Bill suffers from similar problems to existing legislation, namely a lack of specificity and precise statutory framing and definitions, which would avoid analogous pitfalls currently associated with POCTAA and section 530 of the Crimes Act. These gaps tend to promote human interests and overlay law and policy with an anthropocentric bent, a point further illustrated by the way that the defence to section 530 operates, particularly in relation to whether the dog was a pest and whether he was killed in the course of exterminating a pest.

As discussed, above, the Court notes that society relates to the same species of animals in different ways [29]. Indeed, had the dog in question proved to be a companion animal, it is doubtful that the arguments whether he was a pest would have been as protracted [95]. Ultimately, the majority of the Court concluded that the dog was a pest animal but that the defendant did not harm or kill the dog for the purposes of exterminating a pest in accordance with section 530(2)(b) [96]. Simpson AJA disagreed that the dog was a pest animal. Her Honour interpreted “extermination of a pest animal” as a composite phrase and found that the evidence did not demonstrate that dog was a member of the class of animals that could be so characterised and (even if two dogs could constitute a class) the single act of attacking the camel was insufficient to establish that those animals were “pest animals” [35].

Even Bell P, who agreed that the dog was a pest, observed that the notion of eliminating or utterly destroying the dog appeared to be at odds with section 22 of the *Companion Animals Act 1998* (NSW) [33]. While that section permits action against an uncontrolled dog to protect persons and property, if the dog is to be killed, it must be done so, “in a manner that causes it to die quickly and without unnecessary suffering” [97]. The notion of unnecessary suffering, is of course, consistent with a welfare approach, but the anthropocentric leanings of animal welfare means that animals close to humans, such as companion animals, are treated more humanely than animals distanced from humanity’s province of community. This inconsistency occurs notwithstanding the fact that animals, such as companion dogs, wild dogs and pest dogs, belong to the same species and thus experience pain, suffering and pleasure (the basis of animal welfare) in the same way. The fact that humans treat the same species of animal differently, has long occupied the mind of commentators [98,99,100,101,102]. It is particularly problematic when regulators attempt to devise policies for managing pest species that rely on wholescale killing [103].

**Figure 1:**
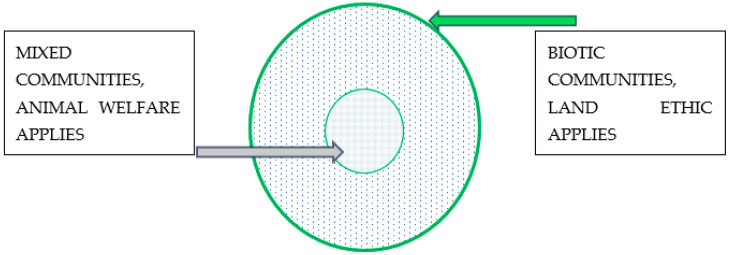
Callicot and Nested Communities.

Callicot attempted to resolve this issue in the 1990s and concluded that the matter is really one of environmental management rather than animal welfare [104]. As shown in Figure 1 below, he argued that society creates “nested communities” with animals. Animals within the human sphere of influence are part of a mixed community of animals and humans, which justifies the application of animal welfare principles. Beyond the mixed community lies the biotic community, which includes other animals, such as wild animals and likely pest animals, where a land ethic applies that justifies the killing of pest animals.

Callicot’s approach has been criticised for falling short of basic moral integrity because it neither provides a sound moral basis for animals within the human community, nor, especially, for animals who are part of the biotic community [105]. The issue continues to be contentious, as evinced by the discussion in the Court of Appeal on whether the dog was a pest and whether it was exterminated, within the parameters of the law. The challenges surrounding these questions demonstrate a fundamental gap in the welfare paradigm, deriving from difficulties the law faces in capturing the many ways humans relate to animals. Moreover, this gap becomes magnified in a legal context as courts grapple with interpretation of unclear and aged legislation.

One such consequence stems from the severity of crimes against animals and the penalties imposed. Section 5 and Section 6 of POCTAA and section 530 of the Crimes Act operate within the same regulatory space. As discussed, section 530 of the Crimes Act applies to serious acts of animal cruelty where the defendant subjectively intends to torture, beat, kill, seriously injure or cause prolonged suffering to an animal. Section 5 of POCTAA prohibits acts of cruelty and section 6 prohibits acts of aggravated cruelty. Definitions of these terms are found in section 4(2) and 4(3) of POCTAA respectively. The former includes acts or omissions which have as a consequence the unreasonable, unnecessary or unjustifiable beating, kicking, killing, wounding, over-loading, over-working, or infliction of pain on an animal. Aggravated cruelty refers to cruelty which results in the death, deformity or serious disablement of an animal or, where the animal is so severely injured, diseased or in such a physical condition that it is cruel to keep it alive. Consequently, with the exception of the requirements for proof beyond reasonable doubt as to the specific intention required in section 530 of the Crimes Act, the conduct elements, or actus reus, of all three offences are the same. And yet, the maximum penalties differ markedly.

The maximum penalty for an offence under section 530 is 5 years imprisonment. Section 530(1A) creates an analogous offence for reckless behaviour where the maximum penalty is 3 years imprisonment. The penalty for a breach of section 5 of POCTAA is 2000 penalty units [106] for a corporation, or 400 penalty units for an individual, who may also be imprisoned for 1 year. The penalty for a breach of section 6 of POCTAA is 5000 penalty units for a corporation or 1000 penalty units for an individual, who may also be imprisoned for 2 years.

Given that section 530 of the Crimes Act operates against the backdrop of other animal cruelty regulation, the penalties themselves potentially create a rather peculiar state of affairs. Arguably the spectrum of offending against animals barely impacts on the Local Court jurisdictional limit under POCTAA of five years’ imprisonment where two or more offences are prosecuted simultaneously. In addition, and in a practical sense, single cases of animal cruelty are rarely prosecuted outside the Local Court, where the two-year jurisdictional limit operates to reduce the effective maximum penalty available. A situation that again demonstrates one of the many gaps in the law’s consideration of animal welfare.

### 3.4. Bridging the Gap—A Good Life for Animals

There are at least two interrelated difficulties in forging a good life for animals from anti-cruelty regulation. First, such regulation largely operates by creating offences once animal cruelty has occurred; and second, this regulation by its very nature can only form part of the animal welfare paradigm. As discussed, animal welfare is a utilitarian ethic that invariably subordinates animal wellbeing to human interests. Undeniably, animal wellbeing, and animal welfare should prohibit cruelty, but the concepts should also maximise the opportunities for animals to live a good life.

Generally speaking, the term “animal welfare” found its way into the literature from the 1960s, and building on this, from the 1990s’ stakeholders began to emphasise the importance of positive states in animals [107]. Animal welfare gained public attention with the publication of Ruth Harrison’s seminal tome, Animal Machines in 1964 [108]. While Harrison herself did not use the term “animal welfare”, the book criticised institutionalised animal cruelty and was instrumental in establishing the Brambell Committee which investigated animal cruelty in intensive animal production in Britain [109]. In 1965, the committee handed down its report, introducing the term “animal welfare” into everyday use [110].

The report described animal welfare as:

both the physical and mental well-being of the animal. Any attempt to evaluate welfare therefore must take into account the scientific evidence available concerning the feelings of animals that can be derived from their structure and functions and also from their behaviour [110].

The report also highlighted the importance of benchmarks such as: freedom from disease, injury, hunger and thirst; provision of sufficient space for animals to move and groom themselves; and, nurturing the ability of animals to express innate behaviour [111]. These benchmarks evolved into the Five Freedoms, a concept that was influential in characterising animal welfare during the last three decades of the twentieth century [111]:

- Freedom from hunger and thirst.
- Freedom from discomfort.
- Freedom from pain, injury and disease.
- Freedom to express normal behaviours.
- Freedom from fear and distress.

Not only were the Five Freedoms highly influential, but they also became widely integrated into popular understandings of animal welfare, ultimately used as a definitional understanding of animal welfare. However, by the mid-1990s, criticism emerged, particularly identifying the lack of meaningful engagement with ethical principles around the fundamental question of whether humans should use animals in the first place [111]. In place of the Five Freedoms, Mellor and others designed a Five Domains Model, to permit structured, systematic animal welfare assessment [112].

Domain theory extends beyond the Five Freedoms by focusing on assessments which are rationally capable of assessing whether welfare has been compromised and then ultimately how to enhance welfare [113]. Possibly because of its foundations in critiquing the Freedoms model, it was not (from the perspective of the developer—David Mellor) designed to become a definition structure for animal welfare. In very brief summary, the Domains model is designed to highlight areas for consideration:

- Internal Domains: focusing on welfare significant internal states:
  ○ Nutrition
  ○ Environment, and
  ○ Health
- External Domains: focusing on welfare significant external circumstances:
  ○ Behaviour
- Once the internal states and external circumstances have been identified, any associated (inferred) affective experiences are accumulated into Domain 5, which is labelled “Mental State”.

It was designed to identify, in a meaningful way, “internal physical/functional states and external circumstances that give rise to negative and/or positive subjective mental experiences (affects) that have animal welfare significance” [113]. Increasingly, the literature acknowledges that anti-cruelty regulation falls short of the mark and best practice “requires that the Five Domains model of animal welfare inform the legislative recognition and definition of animal sentience” [83]. Indeed, without a focus on positive mental and physical states, current animal welfare regimes become bogged down in anti-cruelty regulation that criminalises egregious acts of cruelty, where they are not otherwise, necessary or legally justifiable [83].

In NSW, neither POCTAA, nor s 530 of the Crimes Act mandates minimum standards of care. Accordingly, if animals are to live a life worth living, anti-cruelty regulation needs to undergo transformational change, where at a minimum humans have positive obligations towards animals in accordance with the Five Domains.

## 4. Conclusions

An ordinary member of the public, reading the circumstances of the dog’s death as described in this article and the earlier Riley article, would be forgiven for assuming that the issues for determination by the Court were relatively straightforward. Determining whether a defendant is guilty of an offence of serious animal cruelty, in circumstances where they have admitted to stabbing a dog with a pitchfork, hanging it from a tree, and then hitting it with a mallet, ultimately killing it, would seem simple enough to answer.

Of course, the very “lawyerly” response to that question—“it depends”—is relatively unsatisfactory to most. The “it depends” answer, revolves, in this example, nearly entirely on understandings of statutory interpretation in NSW. Yet the proceedings generated 65 court appearances, including the first Local Court decision, an appeal to the Supreme Court, a further appeal to the NSW Court of Appeal, a second Local Court decision and a second appeal to the NSW Supreme Court. The saga was finally resolved once the defendant withdrew the appeal against his conviction, admitting criminal responsibility for the dog’s death more than seven years after the offence.

The discussion throughout this article on how animal welfare principles are operationalised in criminal law supports arguments for contextual interpretation of animal cruelty provisions. More specifically, the interpretation of regulatory mechanisms, which include legislation, regulations, and codes, should reflect the context of that regulation. Failing to do so raises the likelihood that the criminal law will not be as effective as it might be in positively impacting animals’ lives. Moreover, a lack of contextual interpretation risks limiting the capacity of the law to reduce offending, as offenders may not appreciate the extent to which Parliament has attempted to require minimum standards to improve animals’ lives.

For contextual interpretation to act as a catalyst for improvement, supporting regulation needs, at a minimum, to be clearer about the importance of animal sentience and to extend the protection of the law to a broader range of animals. The recognition of animal sentience is in fact, critical, because otherwise, the law risks legalising cruelty in circumstances of brutal and callous actions against animals. In such cases, the greater the acceptance of animal cruelty, the greater the law’s complicity in widening the welfare gap between the law and animal sentience.

## Figures and Tables

**Table 1:** Precis and chronology: *RSPCA NSW v Brighton*.

Date	Jurisdiction	Outcome
13 October 2017	RSPCA commences proceedings at Campbelltown Local Court	Matter set down for hearing over two days (28–29 June 2019).
28–29 March 2019	Hearing before Magistrate McAnulty Campbelltown Local Court	Matter part heard and adjourned to 17 June 2019.
17 June 2019	Hearing before Magistrate McAnulty Campbelltown Local Court	Brighton found guilty of two sequences of serious animal cruelty.
26 June 2019	Appeal to NSW Supreme Court commenced	Brighton appeal commenced by way of summons in the NSW Supreme Court.
27 June 2019	Hearing before Magistrate McAnulty Campbelltown Local Court concludes	Brighton sentenced to a total term of imprisonment for 3 years and 4 months, with a non-parole period of 2 years and 2 months.
9 September 2019	Appeal to NSW Supreme Court amended	Summons amended to include appeal against the severity of sentence imposed.
3 April 2020	Appeal before Rothman J in the NSW Supreme Court	Oral argument. Decision reserved.
23 April 2020	Rothman J handed down written decision	Appeal allowed.
21 July 2020	Rothman J in the NSW Supreme Court	Decision on costs published.
22 May 2020	Appeal to NSW Court of Appeal, heard by Bell P, Basten JA and Simpson AJA	RSPCA appealed the orders of Rothman J.
8 December 2020	Appeal heard	Judgment reserved.
23 December 2020	Decisions of Bell P, Basten JA and Simpson AJA published	Orders of Rothman J quashed, and the matter was remitted to Local Court for redetermination according to law.
15–17 December 2021	Hearing before Magistrate Degnan Campbelltown Local Court	Judgment reserved.
8 February 2022	Decision of Magistrate Degnan Campbelltown Local Court	Offences proved beyond reasonable doubt.
7 March 2022	Appeal commenced in NSW Supreme Court	Brighton appealed the guilty verdict of the Local Court.
11 July 2022	Sentencing hearing before Magistrate Degnan Campbelltown Local Court	Brighton convicted and sentenced to a total term of imprisonment of two years and two months with a non-parole period of two years.
26 July 2022	Appeal to NSW Supreme Court amended	Summons amended to include appeal against the severity of sentence imposed.
31 May 2023	Appeal listed for hearing before Hamill J	Adjourned on application of the appellant unopposed, leave granted to file further amended summons and submissions on the appeal.
13 December 2023	Hearing in the NSW Supreme Court Hamill J	Brighton withdrew the appeal against conviction. Appeal against sentence upheld, and in lieu of the two-year sentence of imprisonment, a two-year intensive corrections order is imposed as was originally indicated by Justice Rothman.
12 January 2024	Hamill J publishes the judgment.	Appeal against the conviction dismissed, leave to appeal against the sentence granted. Intensive correction order imposed subject to conditions.

**Table 2:** Elements of s530(1) offence provision.

Element	Conduct/State of Affairs	
1. The defendant, with the intention of inflicting severe pain [17]	a. Tortures an animal,	
	b. Beats an animal,	
	c. Commits any other serious act of cruelty on an animal,	serious act of cruelty on an animal includes the act of using the animal as a lure or kill in the manner referred to in section 21 (1) (d) of the *Prevention of Cruelty to Animals Act 1979*.
	And,	
	d. Kills or seriously injures or causes prolonged suffering to the animal,	kill or seriously injure an animal includes, in the case where the animal is used as a lure or kill in the manner referred to in section 21 (1) (d) of the *Prevention of Cruelty to Animals Act 1979*, cause or permit a dog to kill or seriously injure the animal.
Element/Defence?		
2. The defendant does not [18]	a. Perform the conduct in accordance with an Animal Research Act authority, or any other Act or law; or	
	Perform the conduct in the course of or for the purposes of b. routine agricultural or animal husbandry activities, c. recognised religious practices, d. the extermination of pest animals or e. veterinary practice.	

## Data Availability

No new data were created or analyzed in this study.
